# The quality of Medicaid and Medicare data obtained from CMS and its contractors: implications for pharmacoepidemiology

**DOI:** 10.1186/s12913-017-2247-7

**Published:** 2017-04-26

**Authors:** Charles E. Leonard, Colleen M. Brensinger, Young Hee Nam, Warren B. Bilker, Geralyn M. Barosso, Margaret J. Mangaali, Sean Hennessy

**Affiliations:** 1 Center for Pharmacoepidemiology Research and Training, Department of Biostatistics, Epidemiology, and Informatics, Perelman School of Medicine, University of Pennsylvania, 423 Guardian Drive, Philadelphia, PA 19104-4865 USA; 2 Center for Clinical Epidemiology and Biostatistics, Department of Biostatistics, Epidemiology, and Informatics, Perelman School of Medicine, University of Pennsylvania, 423 Guardian Drive, Philadelphia, PA 19104-4865 USA; 3 Division of Health Policy and Management, School of Public Health, University of Minnesota, 420 Delaware Street SE, Mayo D355, Minneapolis, MN 55455-0381 USA; 4 Department of Systems Pharmacology and Translational Therapeutics, Perelman School of Medicine, University of Pennsylvania, 34th Street & Civic Center Boulevard, Philadelphia, PA 19104-5158 USA

**Keywords:** Centers for Medicare and Medicaid Services (U.S.), Data accuracy, Databases as a topic, International Classification of Diseases, Medicaid, Medicare, Pharmacoepidemiology

## Abstract

**Background:**

Administrative claims of United States Centers for Medicare and Medicaid Services (CMS) beneficiaries have long been used in non-experimental research. While CMS performs in-house checks of these claims, little is known of their quality for conducting pharmacoepidemiologic research. We performed exploratory analyses of the quality of Medicaid and Medicare data obtained from CMS and its contractors.

**Methods:**

Our study population consisted of Medicaid beneficiaries (with and without dual coverage by Medicare) from California, Florida, New York, Ohio, and Pennsylvania. We obtained and compiled 1999–2011 data from these state Medicaid programs (constituting about 38% of nationwide Medicaid enrollment), together with corresponding national Medicare data for dually-enrolled beneficiaries. This descriptive study examined longitudinal patterns in: dispensed prescriptions by state, by quarter; and inpatient hospitalizations by federal benefit, state, and age group. We further examined discrepancies between demographic characteristics and disease states, in particular frequencies of pregnancy complications among men and women beyond childbearing age, and prostate cancers among women.

**Results:**

Dispensed prescriptions generally increased steadily and consistently over time, suggesting that these claims may be complete. A commercially-available National Drug Code lookup database was able to identify the dispensed drug for 95.2–99.4% of these claims. Because of co-coverage by Medicare, Medicaid data appeared to miss a substantial number of hospitalizations among beneficiaries ≥ 45 years of age. Pregnancy complication diagnoses were rare in males and in females ≥ 60 years of age, and prostate cancer diagnoses were rare in females.

**Conclusions:**

CMS claims from five large states obtained directly from CMS and its contractors appeared to be of high quality. Researchers using Medicaid data to study hospital outcomes should obtain supplemental Medicare data on dual enrollees, even for non-elders.

**Trial Registration:**

Not applicable.

**Electronic supplementary material:**

The online version of this article (doi:10.1186/s12913-017-2247-7) contains supplementary material, which is available to authorized users.

## Background

United States (US) Medicaid data are widely used for epidemiologic, health services, and policy research [1–3]. Given the potential public health importance of findings arising from such studies, it is critical to understand the quality of the underlying data. Medicaid data are available to researchers via multiple pathways, including from the Centers for Medicare and Medicaid Services (CMS―via its contractors), commercial data vendors, and potentially direct from individual states. Academic, governmental, and non-profit researchers most commonly acquire these data from CMS. In recent years, CMS has made concerted efforts to improve the quality of its raw and research-transformed enrollment and claims files [4, 5]. Their initiatives [4] have led to quality standards, external benchmarking, and publication of file specifications and anomaly reports [6]. While researchers can use these complex technical documents to review validation measures, key summary statistics, and unusual patterns in state data (if documented), there remains a need for a higher-level, overarching examination of data quality.

Researchers often recognize the importance of evaluating the completeness and validity of particular measures of exposure, outcome, and other explanatory factors that will be relied upon in a particular study [7]. Yet, few first examine overarching data quality. Given this, we examined broad indicators of potential error in US Medicaid and Medicare data acquired from CMS and its contractors.

## Methods

Over 14 calendar years (2003–2016), supported by grants from the US National Institutes of Health, we requested and obtained Medicaid Analytic Extract (MAX) files [8] from 1999–2011 (hereafter referred to as file years) for California, Florida, New York, Ohio, and Pennsylvania. We selected these states for study since they are geographically diverse and have a combined prevalent enrollment of nearly 26 million persons, or about 38% of the nationwide Medicaid program [9]. For Medicaid beneficiaries in these states with at least some period of Medicare coverage (i.e., dual enrollees), we further requested and obtained their Medicare claims from the following research identifiable files (RIFs): Medicare Provider Analysis and Review (MedPAR―including short stay hospital, long stay hospital, and skilled nursing facility), Prescription Drug Event (PDE―from Medicare Part D’s 2006 implementation onward), Carrier, and Outpatient [10]. Therefore, the population under study included Medicaid beneficiaries of five large states with and without dual coverage by Medicare. Data were obtained directly from CMS and two different CMS research data distribution contractors over the 14-calendar year period (CMS [Baltimore, Maryland] from 2003–2005, Acumen [Burlingame, California] from 2006–2008, and Buccaneer/General Dynamics [Falls Church, Virginia] from 2009–2016).

We were able to use identifiers provided in the data to track unique beneficiaries longitudinally. Using the MAX Personal Summary file, we first identified beneficiaries without a gap in Medicaid enrollment in a given file year―acknowledging that not all individuals had the same beginning date of their initial enrollment. We then determined the proportion of such beneficiaries without a gap in Medicaid enrollment in each subsequent file year. This served to quantify the persistence of Medicaid enrollment in beneficiaries over long periods of time.

We then graphically summarized several important parameters to assess data completeness and validity. Because our principal use of these data is for pharmacoepidemiologic research, we first looked for unexplained variation in the number of dispensed prescriptions per quarter in each state, which might suggest incomplete prescription data for certain time periods [11]. Relatedly, we also measured the proportion of MAX Prescription and Medicare PDE claims for which the billed National Drug Code (NDC) corresponded to a record in a commercially-available NDC database (Lexicon Plus v.02.01.2016, Cerner Multum: Denver, Colorado). For billed NDCs without a matching record in Lexicon Plus, we used the following alternate sources to identify such products: RxNorm (US National Library of Medicine: Bethesda, Maryland); then state Medicaid drug lists; and then the NDC Directory (US Food and Drug Administration: Silver Spring, Maryland).

We also plotted the ratio of hospitalizations to beneficiary population size in each state, stratified by age group. We did this first using MAX Inpatient data alone, then adding hospitalizations identified by supplementing with Medicare data (MedPAR short stay hospital RIF) to determine the importance of obtaining Medicare data on dual enrollees. To avoid double-counting hospitalizations recorded in both Medicaid and Medicare, we included only one hospitalization per beneficiary per day.

We also examined the frequency of obvious diagnostic miscoding by comparing quarterly counts of claims with a diagnosis of *Complications of Pregnancy, Childbirth, and Puerperium* (International Classification of Diseases, 9th revision, clinical modification [ICD-9-CM] codes 630–677 and subcodes) among females age < 60, females age ≥ 60, and males. Finally, we compared quarterly counts of claims with a diagnosis of prostate cancer (ICD-9-CM: 185, 233.4, 222.2, 236.5, and subcodes) between males and females.

Medicaid and Medicare data access was governed by a data use agreement executed between The Trustees of the University of Pennsylvania and CMS. The University of Pennsylvania’s institutional review board approved the activities described herein.

## Results

Among 15,627,762 beneficiaries identified in file year 1999, 12,649,367 (80.9%) had no enrollment gaps through the year’s end. About 14% of these beneficiaries remained continuously enrolled through the end of 2011―a period of 13 years. Table 1 presents proportions for interim time points and also for beneficiaries newly-identified as having a full year of continuous enrollment post-1999. Raw counts of beneficiaries are presented in Additional file 1: Table S1.

**Table 1:** Proportion of beneficiaries without a gap in Medicaid enrollment in each subsequent file year following their initial file year devoid of a gap, using 1999–2011 Medicaid Analytic Extract Personal Summary files from CA, FL, NY, OH, and PA

	1999	2000	2001	2002	2003	2004	2005	2006	2007	2008	2009	2010	2011
1999	100	58	47	41	35	30	27	23	21	19	17	15	14
2000		100	56	42	31	14	12	10	8	7	6	5	5
2001			100	50	33	21	17	13	11	9	8	7	6
2002				100	48	28	21	17	13	11	10	9	8
2003					100	41	27	20	15	13	11	9	8
2004						100	52	34	26	12	10	9	7
2005							100	44	28	18	15	13	11
2006								100	44	25	20	17	14
2007									100	39	28	22	18
2008										100	56	42	34
2009											100	50	34
2010												100	48
2011													100

**Fig. 1:**
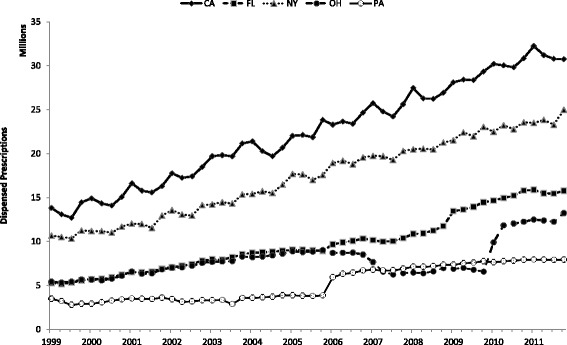
Prescriptions dispensed per quarter per state, from Medicaid Analytic Extract Prescription (1999–2011) and Medicare Part D Prescription Drug Event files (2006–2011)

Figure 1 depicts quarterly prescriptions dispensed per state. Dispensed prescriptions generally increased consistently over time, except for Ohio in which they decreased then plateaued during 2006–2010 (see *Discussion*). The proportion of dispensed prescriptions in which a claim NDC matched a record in Lexicon Plus was 95.2 − 98.4% in MAX Prescription and 99.2 − 99.4% in Medicare PDE data, depending on file year. See Additional file 1: Figure S1 for state-specific trends. Claim NDCs not identified by Lexicon Plus were most commonly for condoms and diabetes care supplies (Additional file 1: Table S2).

**Fig. 2:**
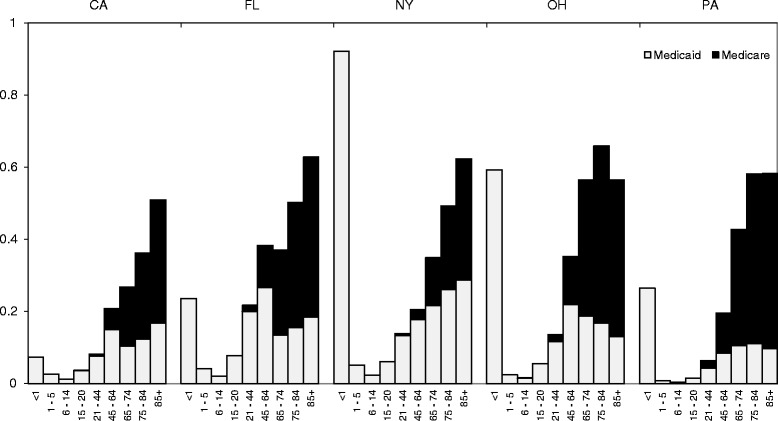
Ratio of number of claims for an inpatient hospitalization to the size of the enrollee population, stratified by age group and state (2011)

Figure 2 depicts the annual rate of hospitalization in each state, stratified by age group and federal benefit, considering Medicaid as primary and Medicare as supplemental. As expected, many hospitalizations in older adults would have been missed if one relied exclusively on Medicaid data. Surprisingly, many hospitalizations in the 45–65 year age group would have been missed if one relied exclusively on Medicaid data. There was a nearly monotonic increase in the rate of hospitalization by age, beginning with the 6–14 year age group.

Additional file 1: Figures S2 and S3 depict quarterly counts of claims with a diagnosis of *Complications of Pregnancy, Childbirth, and Puerperium* and with a diagnosis of prostate cancer, respectively. Encouragingly, there were few pregnancy-related claims in females age ≥ 60 (130,987 [0.2% of pregnancy-related claims]) and in males (197,337 [0.3% of pregnancy-related claims]), and few prostate cancer claims in females (24,839 [0.9% of prostate cancer-related claims]). Instances of apparent miscoding were less common in inpatient than outpatient claims (0.3 vs. 0.4% overall, respectively).

## Discussion

Medicaid and Medicare data provided by CMS and its contractors are widely utilized in epidemiologic, policy, and health services research. However, errors in data can lead to incorrect scientific inferences and evaluations of public policy. Encouragingly, the CMS dataset under study appeared to be of high quality.

This dataset provides one an ability to follow a surprisingly large number of beneficiaries without gaps in Medicaid enrollment over long periods of time, despite a large proportion of beneficiaries under study having at least some period of disenrollment. This is important to researchers wishing to study long-term effects of medical products or policy decisions, for example.

Further, dispensed prescriptions generally increased steadily and consistently over time, suggesting that these claims from the Medicaid programs and file years under study may be complete. An exception may be represented by a dip in Ohio’s prescription claims from 2006–2010, a trend likely driven by managed care expansion [12, 13]. During this time, pharmacy benefits in Ohio were carved-in to managed care and the state was undergoing their Medicaid Management Information System replacement project [14]. Therefore, such claims were not reported to CMS. This explainable “missingness” is in contrast to our prior finding of unexplained variation in prescription claims over time in CMS data obtained from a commercial vendor [11]. We were also encouraged that 95–99% of the prescription claims billed for an NDC identifiable in Lexicon Plus, a commonly-used drug lookup database; this is important since the NDC is the principal method for identifying dispensed drugs in US claims data. That being said, researchers interested in identifying non-drug products (e.g., diabetes supplies) billable to CMS may wish to use an alternative or multiple NDC lookup databases to ensure complete capture.

The pattern of hospitalization rates by age group within Medicaid claims alone is similar to prior findings [11, 15], in which the apparent rate increases up to age 64, then declines at age 65. This implausible pattern is probably an artifact of benefit structures, in which hospitalizations of Medicaid beneficiaries age ≥ 65 who are enrolled in Medicare are covered by Medicare (the primary payer for dual enrollees). Notably, reliance on Medicaid claims alone would have also missed a substantive number of hospitalizations in non-elders, especially among persons age 45–64. This may be due to the fact that non-elders account for ~40% of all dual enrollees and have poorer health than older adults enrolled in Medicare alone [16]. These findings reinforce the importance of obtaining corresponding Medicare claims of dual enrollees in studies using Medicaid data, even if limited to a non-elder adult population [11, 15]. High rates of hospitalization in beneficiaries age <1 year appear to be driven by diagnostic coding of liveborn infant status in newborns.

We examined two crude markers of apparent diagnostic miscoding (i.e., pregnancy complications in males and older females, prostate cancer in females) and found that gross inconsistencies were uncommon. While reassuring, this finding does not eliminate the need to formally evaluate the validity and performance metrics of specific health outcomes of interest. Fortunately, it is now possible, for research purposes, to access primary medical records to validate diagnoses from inpatient and outpatient Medicaid and Medicare claims―with retrieval rates ranging from 29–89% for inpatient charts and 27–66% for outpatient charts [17–27].

We are unaware of a single standard approach to examine the general validity of a health services database. Therefore, we selected metrics that were broadly applicable, intuitively appealing, easy to measure, and easy to interpret. This is consistent with our prior work in this area [15] and in alignment with fit-for-use quality assessment components described by Kahn et al. [28] and Brown et al. [29] The findings herein build upon our prior work by: a) including an additional 11 file years of data, thereby allowing us to examine long-term trends in data quality and quantify the persistence of the Medicaid population; b) including Medicare PDE data, since its implementation in 2006; and c) assessing the consistency in quality across multiple data contractors. While other researchers have examined some broad measures of CMS data quality [30, 31], their datasets under study were from the 1980s and predated the current model by which CMS prepares data for and provides data to researchers.

Big data is a large part of the future of healthcare [32]. However, the use and analysis of big data must be based on accurate and high-quality information―a necessary condition for generating value from big data [33]. Medicaid data available from CMS have tremendous potential utility for research that will ultimately improve the health of the public. Performing exploratory data analyses, such as that conducted herein, is an important first step in using administrative databases. Of course, failure to identify problems in the course of such analyses is no guarantee that the data are valid and complete—especially when selected quality metrics represent a tiny fraction of metrics that could be examined (e.g., trends in claims for ambulatory care encounters, trends in claims for laboratory orders [29]). Given the potential for error in administrative data due to variation in individual states’ program structures and data processing practices, such as diagnostic miscoding, the analyses presented herein can provide a baseline level of understanding of such data.

## Conclusion

In conclusion, we broadly examined the quality of thirteen file years of Medicaid and Medicare data from five large states obtained via CMS and its contractors. The findings are reassuring to researchers―millions of beneficiaries are able to be studied over time without gaps in enrollment, prescription claims appear to be complete and their NDCs identifiable, and obvious diagnostic miscoding is rare. Researchers using Medicaid data to study hospital outcomes should obtain supplementary Medicare data on dual enrollees for studies of persons age 45 years and above.

## Additional file

Additional file 1: Supplemental data consisting of three additional figures and two additional tables. (DOCX 97.2 kb)

## Abbreviations

CMS: Centers for Medicare and Medicaid Services
ICD-9-CM: International Classification of Diseases 9th Revision Clinical Modification
MAX: Medicaid Analytic Extract
MedPAR: Medicare Provider Analysis and Review
NDC: National Drug Code
PDE: Prescription Drug Event
RIF: Research Identifiable File
US: United States
