# Supplementary material for: The quality of Medicaid and Medicare data obtained from CMS and its contractors: implications for pharmacoepidemiology
Source: BMC Health Serv Res. 2017 Apr 26;17:304. doi: 10.1186/s12913-017-2247-7 (PMC5406992; doi:10.1186/s12913-017-2247-7)
Supplement: Additional file 1: — Supplemental data consisting of three additional figures and two additional tables. (DOCX 97.2 kb) [file 12913_2017_2247_MOESM1_ESM.docx]

**ADDITIONAL FILE 1**

**Figure S1**. Proportion of prescription claims for which the billed National Drug Code (NDC) corresponded to a record in the Lexicon Plus NDC database, annually by state.

Panel 1: Medicaid Analytic Extract Prescription file.

Panel 2: Medicare Prescription Drug Event file (Medicare Part D coverage began in 2006).

Please note that the y-axis scales are from 90%–100%.

**Figure S2**. Claims per quarter with a diagnosis of complications of pregnancy, childbirth, and the puerperium, from all encounter file types under study (1999−2011).

Please note that the y-axis is on the log scale.

**Figure S3**. Claims per quarter with a diagnosis of prostate cancer, from all encounter file types under study (1999−2011).

Please note that the y-axis is on the log scale.

The increase in apparently miscoded claims during 2008 was driven by increased coding of *carcinoma in situ* among beneficiaries in Pennsylvania.

**Table S1**. Beneficiaries without a gap in Medicaid enrollment in each subsequent file year following their initial file year devoid of a gap, using 1999–2011 Medicaid Analytic Extract Personal Summary files from CA, FL, NY, OH, and PA.

|  | **1999** | **2000** | **2001** | **2002** | **2003** | **2004** | **2005** | **2006** | **2007** | **2008** | **2009** | **2010** | **2011** |
| --- | --- | --- | --- | --- | --- | --- | --- | --- | --- | --- | --- | --- | --- |
| **1999** | 12,649,367 | 7,318,014 | 5,920,024 | 5,135,963 | 4,400,413 | 3,815,848 | 3,367,283 | 2,956,519 | 2,623,458 | 2,352,147 | 2,135,707 | 1,942,529 | 1,746,314 |
| **2000** |  | 5,838,949 | 3,243,713 | 2,424,550 | 1,826,712 | 842,611 | 688,089 | 562,100 | 469,348 | 404,203 | 358,115 | 320,202 | 283,180 |
| **2001** |  |  | 5,382,732 | 2,704,769 | 1,762,314 | 1,130,670 | 893,031 | 711,893 | 583,384 | 495,065 | 435,964 | 387,991 | 342,438 |
| **2002** |  |  |  | 5,768,931 | 2,794,980 | 1,627,139 | 1,235,087 | 954,234 | 767,065 | 642,688 | 560,762 | 494,686 | 434,424 |
| **2003** |  |  |  |  | 5,934,426 | 2,429,186 | 1,631,063 | 1,181,684 | 912,304 | 747,473 | 643,747 | 561,571 | 488,581 |
| **2004** |  |  |  |  |  | 7,463,631 | 3,855,063 | 2,571,015 | 1,911,890 | 887,428 | 751,959 | 647,782 | 557,215 |
| **2005** |  |  |  |  |  |  | 5,964,883 | 2,606,769 | 1,642,677 | 1,086,269 | 898,172 | 764,871 | 651,627 |
| **2006** |  |  |  |  |  |  |  | 5,827,496 | 2,550,919 | 1,469,791 | 1,162,922 | 965,258 | 808,394 |
| **2007** |  |  |  |  |  |  |  |  | 5,977,892 | 2,359,960 | 1,681,110 | 1,338,521 | 1,093,456 |
| **2008** |  |  |  |  |  |  |  |  |  | 8,004,805 | 4,461,413 | 3,352,240 | 2,697,574 |
| **2009** |  |  |  |  |  |  |  |  |  |  | 6,854,400 | 3,432,700 | 2,362,282 |
| **2010** |  |  |  |  |  |  |  |  |  |  |  | 6,507,273 | 3,131,898 |
| **2011** |  |  |  |  |  |  |  |  |  |  |  |  | 7,181,060 |

**Table S2**. Top 10 National Drug Codes billed for within 1999–2011 Centers for Medicare and Medicaid Services (CMS) data that do not match a record within Lexicon Plus, by CMS claim source

| National Drug Code | CMS claim source* | Dispensings within claim source | % of dispensings among all dispensings without a matching record in Lexicon Plus | Product | Source of product identity** |
| --- | --- | --- | --- | --- | --- |
| 888888888888 | MAX RX | 5,470,442 | 8.7 | N/A*** | CMS MAX Data Anomaly Reports |
| 00536999512 | MAX RX | 2,240,047 | 3.6 | Condoms | Ohio Medicaid Drug List |
| 53885024510 | MAX RX | 1,691,626 | 2.7 | OneTouch Ultra Test Strips | Ohio Medicaid Drug List |
| 53885024450 | MAX RX | 1,269,039 | 2.0 | OneTouch Ultra Test Strips | Ohio Medicaid Drug List |
| 53885039310 | MAX RX | 916,014 | 1.5 | OneTouch UltraSoft Lancet | Ohio Medicaid Drug List |
| 00193654621 | MAX RX | 636,361 | 1.0 | Microlet Lancet | Ohio Medicaid Drug List |
| 50924097110 | MAX RX | 624,542 | 1.0 | Accu-Chek SoftClix Lancet | Ohio Medicaid Drug List |
| 00193708050 | MAX RX | 616,474 | 1.0 | Contour Test Strips | Ohio Medicaid Drug List |
| 08317990950 | MAX RX | 526,598 | 0.8 | Assure Haemolance Plus Lancet | Ohio Medicaid Drug List |
| 24385000726 | MAX RX | 516,643 | 0.8 | Pedia Relief Liq Cgh/Cold | National Association of Chain Drug Stores |
| 08290328468 | Medicare PDE | 571,726 | 8.3 | BD Ultra-Fine II Short Syringe Needle | Ohio Medicaid Drug List |
| 08290328466 | Medicare PDE | 480,937 | 6.9 | BD Ultra-Fine Original Syringe Needle | Ohio Medicaid Drug List |
| 08290320109 | Medicare PDE | 433,588 | 6.3 | BD Ultra-Fine Pen Needle | Ohio Medicaid Drug List |
| 08290320119 | Medicare PDE | 316,505 | 4.6 | BD Ultra-Fine Pen Needle | Ohio Medicaid Drug List |
| 08290328411 | Medicare PDE | 309,836 | 4.5 | BD Ultra-Fine Original Syringe Needle | Ohio Medicaid Drug List |
| 08290328418 | Medicare PDE | 288,058 | 4.2 | BD Ultra-Fine II Short Syringe Needle | Ohio Medicaid Drug List |
| 08290328465 | Medicare PDE | 232,258 | 3.4 | BD Micro-Fine IV Syringe Needle | Ohio Medicaid Drug List |
| 00169185250 | Medicare PDE | 193,706 | 2.8 | Novofine 30 Disposable Needle | Ohio Medicaid Drug List |
| 08290328438 | Medicare PDE | 152,241 | 2.2 | BD Ultra-Fine II Short Syringe Needle | Ohio Medicaid Drug List |
| 08290328410 | Medicare PDE | 141,915 | 2.1 | BD Micro-Fine IV Syringe Needle | Ohio Medicaid Drug List |

MAX = Medicaid Analytic Extract; PDE = Prescription Drug Event; RX = prescription

* MAX RX data from Medicaid beneficiaries of California, Florida, New York, Ohio, and Pennsylvania; Medicare PDE data among dually-enrolled beneficiaries in these states

** CMS MAX Data Anomaly Reports available at: [www.cms.gov/Research-Statistics-Data-and-Systems/Computer-Data-and-Systems/MedicaidDataSourcesGenInfo/MAXGeneralInformation.html](http://www.cms.gov/Research-Statistics-Data-and-Systems/Computer-Data-and-Systems/MedicaidDataSourcesGenInfo/MAXGeneralInformation.html); Ohio Medicaid Drug List National Drug Code lookup available at: [medlist.ohio.gov/main_domain/home.jsf](http://medlist.ohio.gov/main_domain/home.jsf); National Association of Chain Drug Stores lookup available at [www.heraldtribune.com/assets/pdf/SH676171.PDF](http://www.heraldtribune.com/assets/pdf/SH676171.PDF)

*** >99.9% of 12-digit, 8-filled NDCs identified in California MAX RX data―indicative of crossover claims in that state
